# Survivin-specific CD4+ T cells are decreased in patients with survivin-positive myeloma

**DOI:** 10.1186/s40425-015-0065-1

**Published:** 2015-05-19

**Authors:** Frederick L Locke, Meghan Menges, Anandharaman Veerapathran, Domenico Coppola, Dmitry Gabrilovich, Claudio Anasetti

**Affiliations:** Moffitt Cancer Center, Department of Blood and Marrow Transplantation, Tampa, USA; Moffitt Cancer Center, Department of Pathology, Tampa, USA; The Wistar Institute, Tumor Microenvironment and Metastasis Program, Philadelphia, USA

## Abstract

**Background:**

Survivin is a small protein inhibitor of apoptosis and a tumor associated antigen. Survivin expression in multiple myeloma is associated with poor prognosis, disease progression, and drug resistance. The CD4+ response against survivin remains uncharacterized.

**Methods:**

In order to better understand the anti-tumor immune response to survivin, and optimize vaccination strategies, we characterized the spontaneous CD4+CD25- T cell response against survivin in healthy donors and myeloma patients using survivin derived peptide pools.

**Results:**

Healthy donors and myeloma patients’ CD4+CD25- T cells exhibited a proliferative and IFN-gamma response against survivin peptides loaded onto autologous dendritic cells. We employed limiting dilution analysis to quantify the precursor frequency of survivin reactive CD4+CD25- T cells. Multiple myeloma patients (range 0% to 2.2x10^-3^%, n=12) had fewer survivin reactive CD4+CD25- T cells than healthy blood donors (range 1.1x10^-3^ to 8.4x10^-3^%, n=10), p = 0.021. The survivin reactive CD4+CD25- T cell precursor frequency was inversely associated with tumor survivin mRNA expression (p = 0.0028, r = −1.0, n = 6), and survivin tumor protein expression by IHC (p = 0.0295, r = −0.67, n = 10). A full length mutant survivin protein-pulsed dendritic cell vaccine expanded survivin reactive CD4+CD25- T cells after 12 days of in vitro culture (range 0-540x,median = 42x), and expansion was achieved even in patients with low baseline survivin reactive CD4+ precursors.

**Conclusions:**

We have, for the first time, quantified the circulating CD4+CD25- precursor frequency against survivin and demonstrated this is lower in myeloma patients than healthy donors. The number of survivin reactive CD4+CD25- T cells is inversely associated with tumor survivin expression suggesting suppression of survivin responsive CD4+CD25- T cells. Further exploration of a full length mutant survivin protein vaccine which expands survivin reactive CD4+ cells independent of the survivin reactive precursor frequency is warranted.

**Electronic supplementary material:**

The online version of this article (doi:10.1186/s40425-015-0065-1) contains supplementary material, which is available to authorized users.

## Background

Survivin is a small protein and tumor associated antigen expressed in multiple myeloma. Survivin normally functions as an apoptosis inhibitor, via spindle microtubule and mitotic checkpoint regulation [1]. It is a potential target for immunotherapy since it is highly expressed in many cancers [2-4], it is linked to worse prognosis in both solid and hematologic tumors, and it is undetectable in almost all normal adult tissues [5]. Survivin is overexpressed in myeloma cell lines and its expression in primary myeloma cells is associated with poor prognosis, disease progression, and drug resistance [6,7].

CD8+ T cells specific for survivin have been demonstrated in myeloma patients [8], and survivin-specific CTL responses were generated *in vivo* in tumor-bearing mice [9-11]. For malignant melanoma patients receiving a MHC class I restricted peptide vaccine against survivin, both response to therapy and overall survival were associated with a CD8+ T cell response against survivin [12]. Our present knowledge of human immune response against survivin is almost entirely based upon the induction of cytotoxic CD8+ T cell responses using vaccines or clonotype analysis using single HLA-Class I peptides. Little is known about important CD4+ helper T cell responses against survivin, which are essential for an optimal anti-tumor immune response [13,14]. Cancer patients can have survivin specific CD4+ T cells [15-17] and robust CD4+ responses may be generated with survivin HLA-class II restricted peptide vaccines in cancer patients [18,19]. CD4+ T cells can reject tumors in the absence of CD8+ T cells [20] and provide primary anti-tumor immune responses important for immunosurveillance [21]. The spontaneous CD4+ response against survivin in myeloma patients has not been characterized, and must be understood to optimize vaccine strategies against aggressive survivin expressing myeloma.

Prior evaluation of T cell immune responses against survivin, and most therapeutic survivin cancer vaccines, has relied upon identification of T cells specific for HLA restricted peptides. This strategy has several limitations. Many peptides can be generated from the entire protein. Each peptide is restricted by one or few HLA molecules for presentation to immune cells and HLA molecules are encoded by 15 distinct genes that are the most polymorphic in the entire genome. Therefore, because HLA genes vary widely among people, the probability of one peptide inducing an immune response is low and the breadth of the response is extremely narrow. Survivin derived peptide pools can overcome these limitations and allow study of the immune response against survivin [22]. In order to better understand the survivin specific immune response and optimize vaccination strategies against myeloma, we sought to characterize the survivin specific CD4+ T cell response using survivin derived peptide pools.

## Results

### Human CD4+ T cells exhibit a survivin specific response

**Figure 1:**
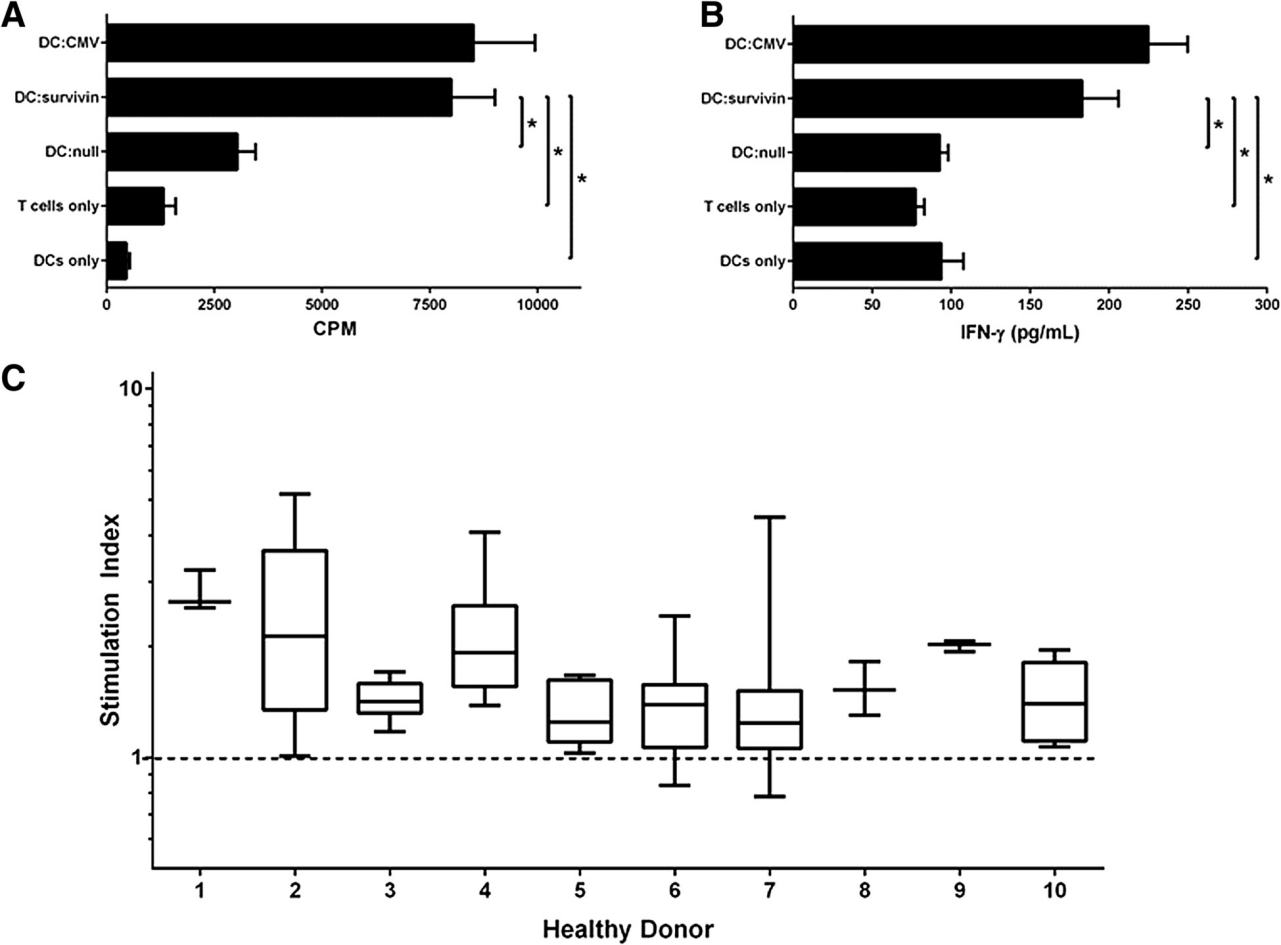
Healthy donor CD4+CD25- T cells proliferate and secrete IFN-gamma in response to survivin peptide pools. 1×10^5^ Purified CD4+CD25- cells were stimulated for 6 days with 1 ×10^4^ autologous DCs loaded with CMV peptide pool (DC:CMV) positive control, survivin peptide pool (DC:survivin), or unloaded (DC:null) negative control. Proliferation **(A)** and IFN-gamma secretion **(B)** were elicited by DC:survivin, figures represent the mean of three independent experiments from different healthy donors and error bar represents standard error of the mean. For 10 consecutive evaluable healthy donors, a stimulation index was calculated **(C)** [1×10^5^ CD4+CD25- T cells stimulated with 1×10^4^ DC:survivin (numerator)/Mean of > =10 DC:null stimulated T cell controls (denominator)]. Box and whiskers represents multiple stimulation indices (Line = Mean, Box = 25%-75% CI, Whiskers = minimum and maximum) for each donor. *p < 0.05.

The response of unprimed conventional human CD4+ T cells against survivin was evaluated by quantifying proliferation and IFN-gamma cytokine release against a peptide pool (JPT) derived from survivin. Because the peptides are not restricted to a single HLA type , testing of human T cells does not require HLA typing and stratification since the likelihood of detecting a response is magnified by the pool of peptides. CD4+CD25- T cells from healthy donors proliferated in response to survivin peptide pools loaded onto autologous monocyte derived dendritic cells, similar to responses against common viral antigens (Figure 1A). After 6 days of co-culture, IFN-gamma was detectable within the supernatant (Figure 1B). To evaluate the reactivity of healthy donor CD4+CD25- T cells against survivin we determined the stimulation index for 10 consecutive healthy donors (3–12 replicates per donor). CD4+ proliferative response against survivin was detectable in all 10 healthy donors tested (Figure 1C). Not every well containing 100,000 CD4+ cells exhibited proliferative responses exceeding the unloaded autologous DC response, indicating that these cells are rare. When CD25 cells were not removed, total CD4+ responses were minimal, possibly due to the presence of Tregs (Additional file 1: Figure S1A-B). CD4 proliferative responses against survivin were not different in the presence of CD8 cells (Additional file 1: Figure S1C).

### Limiting dilution analysis quantifies the frequency of survivin reactive CD4+ T cells

**Figure 2:**
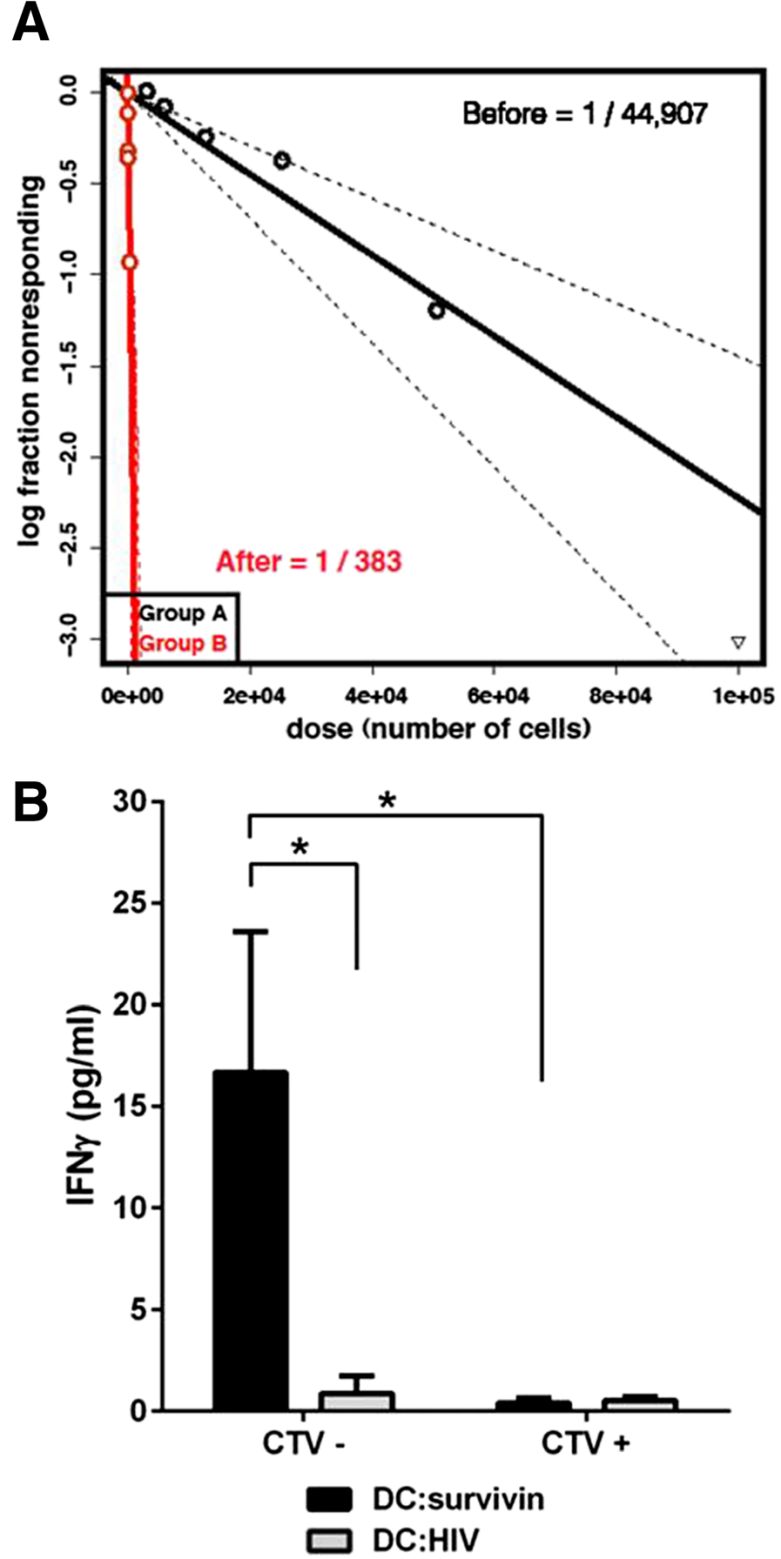
Limiting dilution analysis (LDA) determines the frequency of survivin reactive CD4+CD25- T cells. Serially diluted, purified CD4+CD25- T cells were stimulated with autologous DC:survivin in the presence of exogenous IL-2. Each LDA was performed with a minimum of 10 replicates per cell concentration. **(A)** Log-fraction plot of the LDA of survivin specific CD4+CD25- T cells both before and after expansion with DC:survivin. The slope represents log-active cell fraction, bold lines represent frequency estimates, and non-bold lines show 95% CIs based on the likelihood ratio test of single-hit model. One out of 44,907 cells were estimated to respond to the survivin peptide pool before expansion (Before). Separately, CD4+CD25- T cells from the same donor were expanded using DC:survivin and exogenous IL-2 for 12 days. Cells were collected, enumerated, and rested without cytokines for 2 days. Repeat LDA after expansion demonstrated enrichment for survivin specific T cells to 1 out of 383 (After), p < 0.0001. Representative of 2 independent experiments from separate healthy donors. **(B)** LDA was validated by labeling un-stimulated CD4+CD25- T cells with CTV (cell trace violet) prior to expansion with DC:survivin and exogenous IL-2. After 12 days, T cells were flow sorted into CD4+CTV- (replicated) and CD4+CTV+ (non-replicated), then rested for 2 days without cytokines. 2.5×10^3^ CD4+ T cells were stimulated with DC:survivin or DC:HIV (irrelevant) peptide pool for 24 hours. * = p < 0.05 by t test, error bars indicate the standard deviation. Representative of two independent experiments from separate healthy donors.

To quantify the precursor frequency of survivin specific T cells we performed limiting dilution analysis (LDA) of CD4+CD25- T cells against a fixed dendritic cell concentration (Figure 2A, Before). To validate that the proliferation measured in the LDA was indeed due to reactivity specifically against survivin, CD4+CD25- T cells were concurrently expanded, under the same conditions, for 12 days. Repeat LDA showed the frequency of survivin reactive T cells (ie: % reactive against survivin) in culture increased approximately 100 fold (Figure 2A, After), and the fold expansion of survivin reactive CD4+ cells was 200x. We next confirmed that T cells expanded using DC:survivin were able to secrete IFN-gamma in response to survivin. Before initial DC:survivin stimulation, CD4+CD25- cells were labeled with the proliferative marker cell trace violet (CTV). Following 12 days of expansion with DC:survivin, CD4+ cells were flow sorted into CTV- (replicated) and CTV+ (non-replicated) populations. Replicated T cells showed significant secretion of IFN-gamma (Figure 2B) upon re-challenge with DC:survivin as compared to DCs loaded with an irrelevant HIV protein peptide pool (DC:HIV). Non-replicated CD4+ T cells did not respond to either peptide pool stimulus.

### Multiple myeloma patients have fewer survivin reactive CD4+ T cells than healthy blood donors

**Figure 3:**
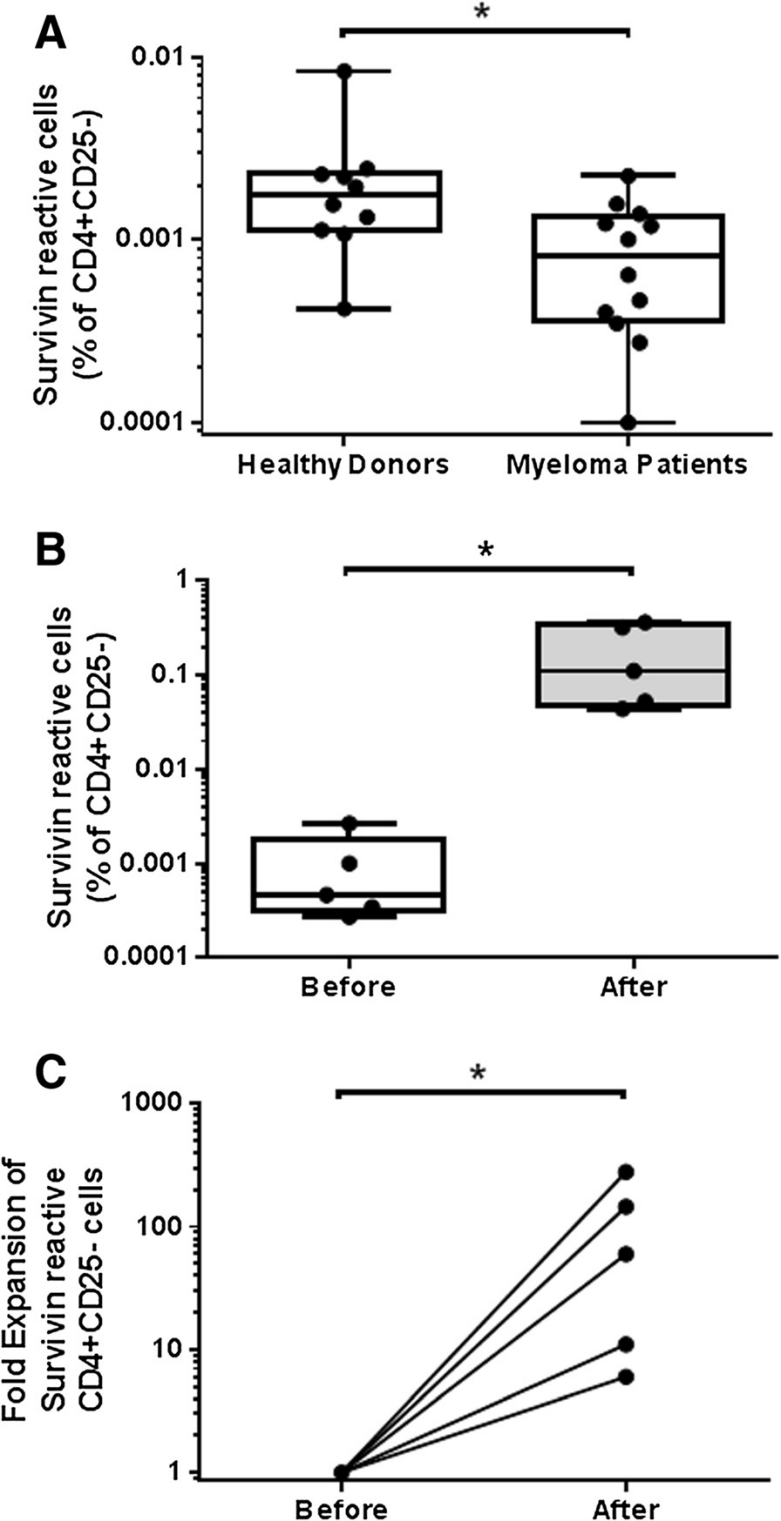
Myeloma patients harbor survivin specific CD4+CD25- T cells which respond to survivin peptide pool loaded autologous DCs. **A.** The survivin reactive CD4+CD25- precursor frequency for 12 consecutive myeloma patients was determined. Myeloma patients survivin reactive cells, as a % of total CD4+CD25- cells were less than that of consecutive healthy donors (p = 0.02, non-parametric t-test). **B-C**. Myeloma patient CD4+CD25- cells were expanded using DC:survivin as described. The survivin reactive CD4+ cell frequency **(B)** was significantly increased; and significant fold expansion of survivin reactive CD4+ cells was observed **(C)**, (before expansion # of survivin reactive cells normalized to 1). Line = Mean, Box = 25%-75% CI, Whiskers = minimum and maximum, *p < 0.05 by non-parametric t-test.

It was previously shown that myeloma patients can harbor CD4 and CD8 T cells reactive against survivin, however this required multiple stimulation and expansion steps, precluding a precise quantification of the circulating T cell precursor frequency. We evaluated the precursor frequency of survivin reactive CD4 T cells in the peripheral blood of 10 consecutive healthy donors and 12 consecutive multiple myeloma patients. Myeloma patients had a significantly lower precursor frequency of survivin reactive CD4+CD25- cells (range 0% to 2.2x10^-3^%) compared to healthy donors (range 1.1x10^-3^ to 8.4x10^-3^%) (Figure 3A). All myeloma patients had received prior therapy, and clinical characteristics are presented in Table 1. Similar to healthy donors, myeloma patient CD4+CD25- T cells expand in response to DC:survivin stimulation (Figure 3B-C). To exclude generalized immune suppression as the cause for the different precursor frequencies, we calculated the stimulation index of T cells stimulated with DCs loaded with a peptide pool derived from common infectious antigens. Results for healthy donors (n = 11, median stimulation index = 2.9) and myeloma patients (n = 7, median stimulation index = 2.7) were similar (Additional file 2: Figure S2A). Because our assay measures proliferation at six days, we considered that myeloma patients may harbor CD4+ effectors which could proliferate at different kinetics than less antigen experienced CD4+ cells within healthy donors. At 72 hours, myeloma patients CD4+CD25- T cell proliferation and IFN-gamma release in response to DC:survivin was not different from healthy donors (Additional file 2: Figure S2B-C). Alternatively, the differentiation state of the survivin reactive CD4+ T cells we identified in myeloma patients could be unprimed naïve cells or antigen experienced memory cells, however the paucity of the cells (Table 1) preclude direct phenotypic evaluation of maturation markers. To evaluate the ability of our assay to capture naïve T cell responses against survivin, we next verified that the peptide loaded self-DCs expressed the co-stimulatory molecules required to activate naïve T cells (Additional file 3: Figure S3A-D). Finally, we verified that our LDA assay was able to detect naïve CD4+ T cell responses in myeloma patients (n = 2) and a cord blood donor unit by stimulating CD4+CD25-CD45RO- cells with autologous DCs loaded with a peptide pool derived from the Consensus B gag motifs of HIV. Since myeloma patients were HIV negative by serology and nucleic acid testing, they would not have primed T cells against HIV derived peptides [23], and similarly for cord blood T cells as they are antigen-inexperienced. The HIV reactive precursor frequency was low, yet detectable (Additional file 3: Figure S3E).

**Table 1:** **Patient Characteristics, Tumor Survivin Expression, and CD4+ Survivin Specific Precursor Frequency**

**Age**	**Disease Characteristics**	**Durie Salmon Stage**	**Prior Regimens**	**Imid Therapy**	**IMWG Response**	**High Risk**	Survivin IHC*	Survivin mRNA**	**Survivin Specific CD4+ Precursor Frequency (% of CD4)**
67	IgG lambda	IIB	1	yes	VGPR	no	7	-	0.000000
46	IgG lambda	IIIA	1	no	PR	no	-	-	0.000000
69	IgG kappa	IIA	1	yes	PR	no	8	-	0.000052
67		IIIA	2	yes		yes	5	1.375	0.000274
64	IgG kappa	IA	2	no	VGPR	yes	5	-	0.000346
49	IgG lambda	IIIB	1	yes	VGPR	no	-	-	0.000349
56	IgG kappa	IIIB	2	no	PR	no	7	1.248	0.000401
46	IgA kappa	IA	2	no	PR	no	4	0.814	0.000466
57	IgG kappa	IIIB	2	yes	PR	no	6	0.625	0.000643
69	IgG kappa	IIIA	1	yes	PR	yes	-	-	0.000798
69	IgG kappa	IA	1	no	VGPR	no	-	-	0.001007
62	IgG lambda	IIIA	1	no	PR	no	-	-	0.001195
66	IgG kappa	IA	2	no	PR	yes	4	0.481	0.001579
74	IgA lambda	IIIB	2	yes	PR	yes	2	0.226	0.002250
62	IgG kappa	IIIA	1	yes	VGPR	yes	5	-	0.002657
50	lambda light chain	IIA	1	no	VGPR	no	-	-	0.006804

*Survivin IHC of bone marrow plasma cells by the Allred scoring system. **Survivin mRNA of isolated CD138+ marrow cells normalized to GAPDH.

### Multiple myeloma tumor survivin expression is inversely associated with survivin reactive CD4+ T cell frequency

**Figure 4:**
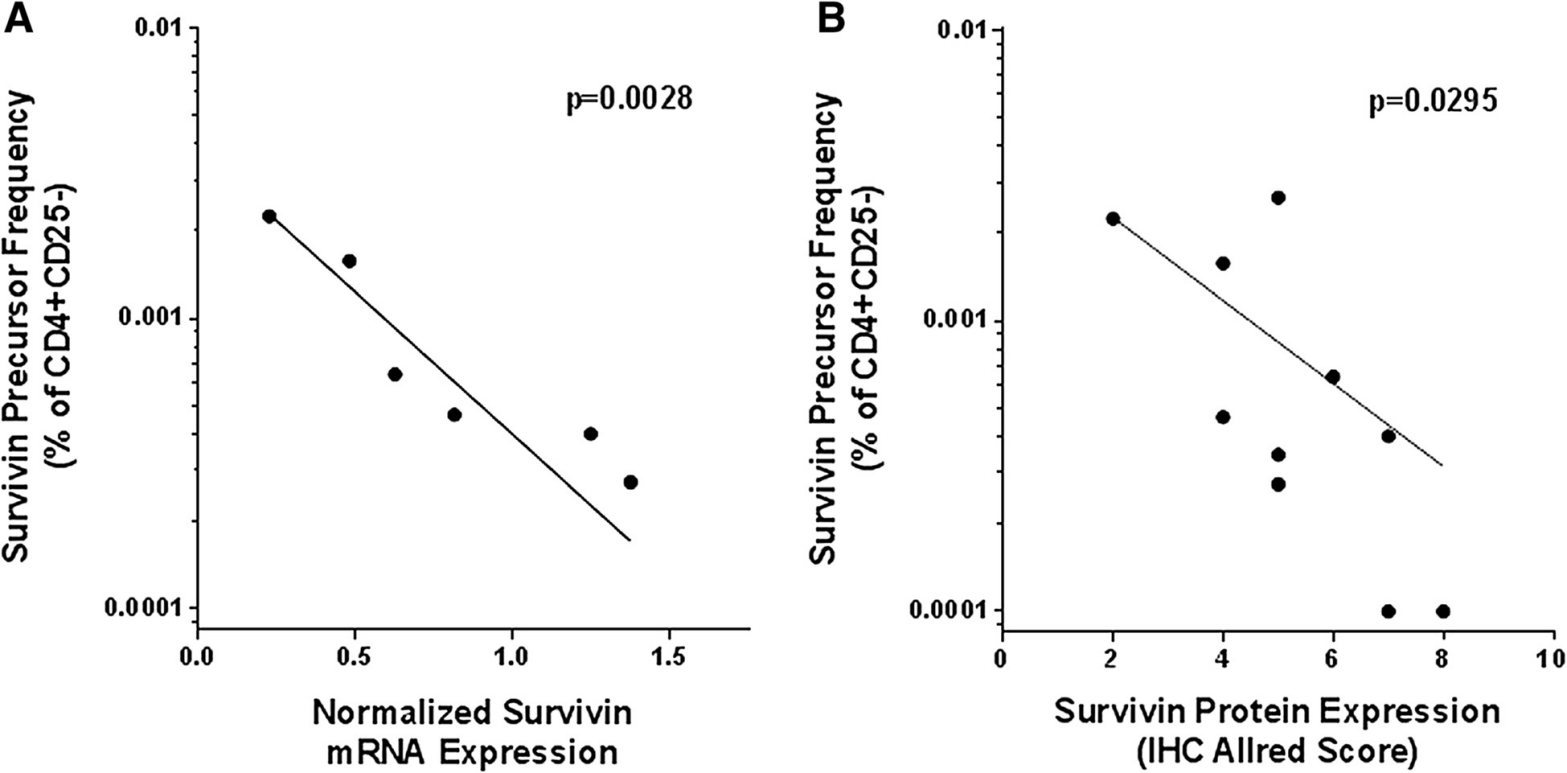
Myeloma tumors express survivin mRNA and protein which negatively correlate with survivin reactive CD4+ T cell frequency. **A.** mRNA expression negatively correlates to the de-novo survivin reactive CD4+CD25- T cell precursor frequency as calculated by LDA. (p = 0.0028 and r = −1.0 by Spearman nonparametric correlation analysis). B. Survivin protein expression, evaluated by the Allred score [46], negatively correlates to the de-novo survivin reactive CD4+CD25- T cell precursor frequency as calculated by LDA. (p = 0.0028 and r = −1.0 by Spearman nonparametric correlation analysis).

CD138+ primary multiple myeloma cells were isolated from patients’ bone marrow aspirates and evaluated for survivin mRNA transcripts (Table 1). The survivin protein expression of myeloma cells within bone marrow biopsy specimens was quantitatively scored based upon immunohistochemistry (Table 1). There was an inverse association between a patient’s survivin reactive CD4+CD25- precursor frequency and their tumor’s survivin expression by quantitative PCR (Figure 4A). Similarly there was an inverse association between survivin protein staining and a patient’s survivin reactive CD4+CD25- precursor frequency (Figure 4B). To evaluate whether CD4+ cells were preferentially trafficking to the bone marrow [24], we compared the peripheral blood and marrow survivin reactive CD4+ T cell precursor frequency in 3 paired samples (n = 3) and did not detect a significant difference (Additional file 3: Figure S3F).

### A full length survivin protein vaccine elicits CD4+ T cell responses in myeloma patients despite low baseline survivin reactive CD4+ precursor frequencies

**Figure 5:**
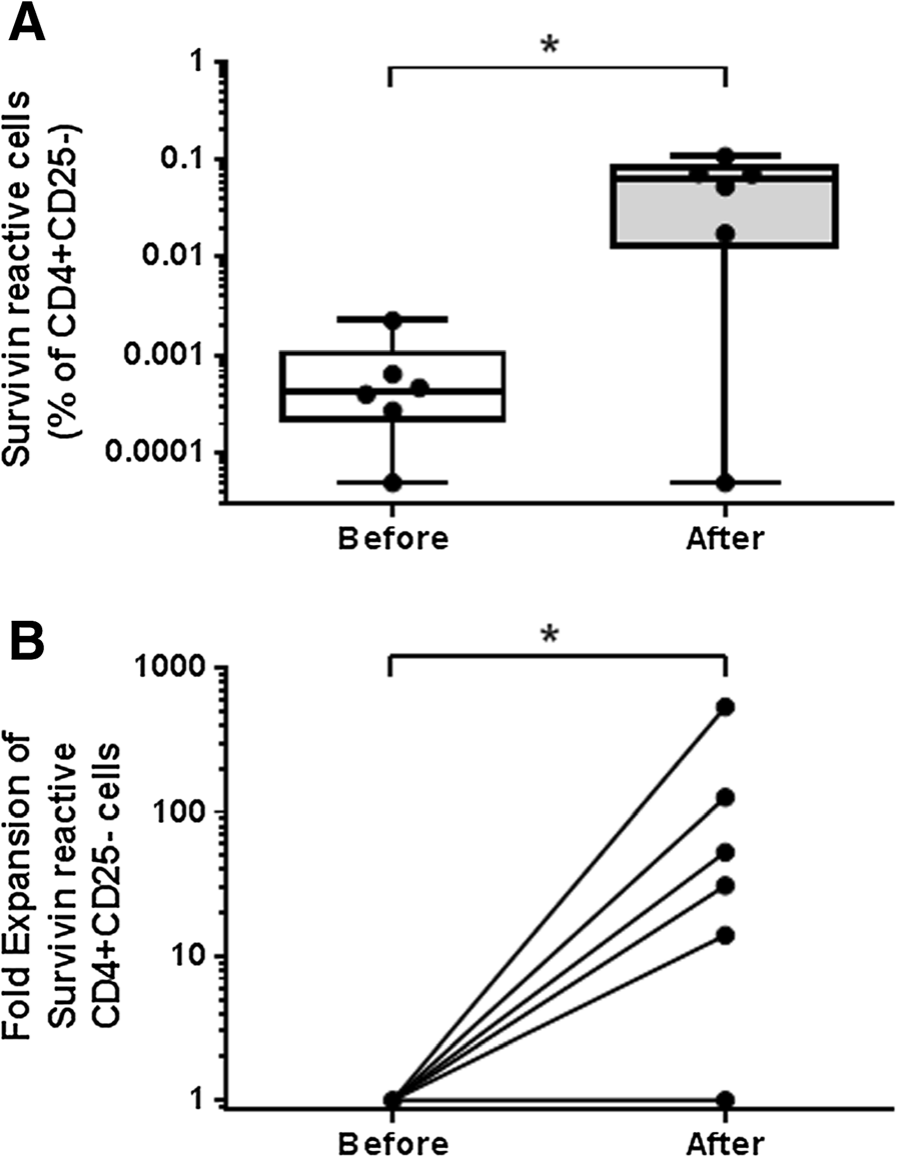
A full length survivin protein vaccine expands survivin specific CD4+ cells even in patients with a low survivin reactive precursor frequency. **A-B.** An adenoviral construct was used to infect autologous myeloma patient DCs which leads to expression of a full length mutant survivin protein. Patient CD4+CD25- T cell survivin reactive frequency was calculated by LDA before and after 12 day co-culture with DC:ad-ms. The survivin vaccine increases the frequency of survivin reactive CD4+ cells **(A),** and significant fold expansion of survivin reactive CD4+ cells was observed **(B)**, (before expansion # of survivin reactive cells normalized to 1). Line = Mean, Box = 25%-75% CI, Whiskers = minimum and maximum, *p < 0.05 by non-parametric t-test. * = p < 0.05 by paired ratio t-test.

We tested the ability of a full length survivin protein vaccine to expand myeloma CD4+CD25- T cells that are reactive against survivin peptide pool loaded autologous DCs. The previously characterized vaccine consists of an adenoviral construct (Ad-ms), which upon infection of autologous DCs (DC:Ad-ms), leads to expression and antigen presentation of a mutated survivin protein [25]. This approach allows for preservation of multiple epitopes, which upon DC antigen presentation are more likely to capture and expand survivin reactive T cells than single or oligo-peptide survivin vaccines. The survivin reactive frequency was determined before and after myeloma patient CD4+CD25- T cells were co-cultured with autologous DCs infected with Ad-ms. Survivin reactive CD4+ T cell frequency was increased after co-culture (Figure 5A). Fold expansion ranged from 0-540×, median = 42× (Figure 5B). The vaccine was able to expand survivin reactive cells even from myeloma patients with a low pre co-culture survivin specific precursor frequency (near or below the limit of detection of the LDA assay), and the survivin reactive precursor frequency of CD4+CD25- cells was not predictive of the fold expansion of survivin reactive with vaccine stimulation (r = −0.4857, p = 0.36 by Spearman correlation analysis).

## Discussion

In healthy donors and myeloma patients we have quantified the peripheral blood CD4+ T cell precursor frequency reactive against survivin [26]. Survivin expression in myeloma patients’ tumors correlates with decreased survivin reactive CD4+ T cells. A full length mutant survivin protein vaccine induces CD4+ responses independent of the survivin specific CD4+ precursor frequency. Our results have several implications.

Healthy donors have detectable T cells responsive against survivin which do not require *in vitro* expansion or cloning techniques to quantify. Prior reports employed methodology including multiple priming and stimulation steps to grow out clonal cells against survivin or vaccination to promote expansion *in vivo*. The quantifiable presence of survivin reactive T cells in healthy donors is not entirely surprising since protein expression of the tumor associated antigen survivin is limited in adult tissues [1,5]. Others have demonstrated CD8+ T cells reactive against survivin in the peripheral blood of chronic lymphocytic leukemia and neuroblastoma patients, and within tumor infiltrating lymphocytes of melanoma patients. Contrary to our finding with CD4+ cells, those CD8+ responses were decreased or absent in healthy donors, although those flow and IHC methods were significantly less sensitive than our precursor frequency assay [27,10,28,15]. The characterization of CD8+ T cell responses against survivin in multiple myeloma patients is a subject of future investigation. We clearly demonstrate CD4+ cells against survivin are circulating within healthy individuals and myeloma patients. The degree to which survivin specific T cells are prone to immune-selection during thymic development is unknown.

Myeloma patients have decreased survivin specific CD4+ cells compared to healthy donors, yet CD4+ responses against common viral antigens were similar. The expression of tumor survivin inversely correlates to the magnitude of the survivin specific T cell precursor frequency. Proliferation against survivin loaded DCs was minimal for both myeloma patients and healthy donors at three days indicating that myeloma patients do not simply harbor primed survivin specific T effectors with different proliferation kinetics. Our assay is able to detect antigen inexperienced cells, as evidenced by the detection of HIV antigen responses in HIV negative individuals and a cord blood unit. Whether the survivin specific CD4+ T cells are naïve or central memory is undetermined. Future experiments could evaluate the survivin reactive precursor frequency of isolated naive, CD4+CD25-CD45RO- T cells, although the number of PBMCs needed to conduct such an experiment is extraordinary. Regardless of their maturation state, clearly these T cells are unsuccessful at mediating spontaneous tumor rejection.

Numerous mechanisms might suppress or delete these cells. The T cells themselves may be exhausted or senescent. Marrow and peripheral blood CD4+ survivin specific precursor frequencies were similar in a subset of MM patients so it unlikely the differences are due to differential trafficking. Active suppression by regulatory T cells (Tregs) [29-31], myeloid derived suppressor cells (MDSC) [32-34], or the tumor microenvironment [35-38] are possible. Expression of inhibitory ligands on tumor [39] or negative regulatory receptors on T cells [40,41] may also play a role in T cell dysfunction against TAAs. These survivin specific T cells are extremely rare and quantifying their precursor frequency is part of the novelty of our results. Because of the paucity of these cells we could not directly test the cells and evaluate the hypothesis they are exhausted, senescent, or subject to negative regulation. Increasing these cells in patients, via vaccination, could evaluate the relevance of survivin specific CD4+ T cells in myeloma patients.

The use of a full length mutant survivin vaccine allows expansion of survivin specific CD4+ T cells regardless of the precursor frequency of the reactive CD4+ survivin cells. The survivin protein vaccine utilizes DCs transduced with an adenoviral construct containing a full-length mutant survivin [25]. This vaccine elicits a survivin-specific immune response when used *in vitro* to stimulate cells from healthy donors and prostate cancer patients [42]. In murine models this vaccine generates a potent multi-epitope survivin-specific response and mediates both *in vitro* and *in vivo* antitumor immunity [43]. A full length protein vaccine is desirable for several reasons. It does not require HLA typing, thereby maximizing patient eligibility. Presentation of multiple antigens will maximize immunogenicity and CD4+ and CD8+ immune responses should be allowable. We have demonstrated here that the vaccine is able to expand CD4+ T cells reactive against survivin. Importantly this is possible regardless of the precursor frequency of survivin reactive T cells. This implies that the vaccine may induce survivin specific CD4+ immune responses even when the survivin specific CD4+ T cell precursor frequency is below the level of detection of our current assay.

An examination of the weakness of our studies reveals that several questions about survivin specific T cell responses for myeloma patients remain to be answered. First, using peptide pools exclusively it is not possible to determine which epitope is inducing given responses. The responses we detect may be monoclonal or oligoclonal. Future investigations using each peptide of the survivin pool separately are being considered. Second, our methods capture functional responses against survivin. It is possible that survivin specific T cells exist in higher numbers but are non-functional. Third, our methodology utilized the depletion of CD25 cells in order to negate Tregs. It is possible Tregs are present and blunting immune responses *in vivo* or within the tumor microenvironment. Furthermore, if survivin specific effector T cell responses are ongoing these cells may, in part, be removed via CD25+ cell depletion. Lastly, the CD8+ cell frequency needs to be quantified in a similar manner to what we demonstrate here for CD4+ cells.

## Conclusions

Accurate methodology and understanding of the CD4+ T cell response against survivin is necessary for the development of cancer immunotherapies against survivin expressing tumors. We have, for the first time, quantified the circulating, CD4+ precursor frequency against survivin in healthy donors and myeloma patients. This response is lower in myeloma patients compared to healthy donors, and inversely correlates to tumor survivin expression. Due to the paucity of these cells, direct evaluation of the mechanisms which blunt the response is not testable. A vaccine strategy to increase these CD4+ T cells in patients would be the ideal strategy to evaluate their activity against myeloma. The use of a full length mutant survivin vaccine expands survivin reactive CD4+ cells independent of the survivin reactive CD4+ precursor frequency.

## Methods

### Sample collection

Healthy donor blood samples were provided as buffy coats from One Blood (St. Petersburg, Florida). Multiple myeloma patient blood and bone marrow aspirate was collected at Moffitt Cancer Center (Tampa, Florida) after informed consent to a sample collection study approved by the University of South Florida Institutional Review Board (MCC 16617). Mononuclear cells were isolated from blood and bone marrow using density gradient centrifugation over Ficoll-Paque PLUS (GE Healthcare, Little Chalfont, UK).

### Tumor cell isolation

CD138+ plasma cells were selected from mononuclear bone marrow cells of multiple myeloma patients using magnetic column separation. Cells were incubated with CD138+ microbeads (Miltenyi, Germany) for 15 minutes, washed and eluted over an MS or LS column (Miltenyi). CD138+ cells retained in the column were collected and purity of >90% was verified by flow cytometry.

### Dendritic cell generation and peptide pool loading

Dendritic cells (DCs) were generated by suspending 7–11×10^6^ PBMCs/mL in serum-free XVIVO-15 media (Lonza, Allendale, NJ) followed by 3 hour culture in a 25 cm^2^ cell culture flask (Corning, Corning, NY). Cells were then washed twice in PBS to remove non-adherent cells. Adherent cells were cultured in serum-free X-VIVO media supplemented with 1000 units/ml each of GM-CSF and IL-4 (R&D Systems, Minneapolis, MN) for six days. DCs were then collected, washed and counted. DCs were loaded with the indicated peptide pool by incubating for one hour at 37°C in 100 μl XVIVO-15 media supplemented with 1 μg/peptide/ml then used for experiments. Pepmix™ Peptide pools were synthesized by the manufacturer (JPT, Germany). The survivin peptide pool consists of 33 peptides derived from a peptide scan (15 mers with 11 aa overlap) through Baculoviral IAP repeat-containing protein 5 (Survivin). Positive control peptide pools included CEFT MHCII (14 peptides each corresponding to a defined HLA class II restricted T-cell epitope from Cytomegalovirus, Epstein-Barr virus, Influenza virus or Clostridium tetani) and HCMVA (pp65) sourced from the 65 kDa lower matrix phosphoprotein of human cytomegalovirus (strain AD169). The HIV-1 peptide pool (123 peptides selected from Con B gag motifs of HIV), or vehicle only, were used as negative controls as indicated.

### T cell isolation

For all experiments, CD4+CD25- T cells were isolated by using magnetic beads from a CD4 negative selection kit supplemented by CD25+ microbeads per the manufacturer’s protocol (Miltenyi). PBMCs were incubated for 10 minutes with a biotin-antibody cocktail including CD8, CD14, CD15, CD16, CD19, CD36, CD56, CD123, TCR γ/δ, and CD235a (Glycophorin A) followed by a 15 minute incubation with both anti-biotin and anti-CD25 microbeads. Labeled cells were passed through an LS column and the negative fraction was collected for use in T cell assays.

### Proliferation assay

[^3^H]thymidine incorporation assay was performed as previously described [44]. Briefly, CD4+CD25- T cells were suspended in X-VIVO-15 (Lonza) media supplemented with 10% human serum (SeraCare, Milford, MA) and penicillin/streptomycin, then seeded into a 96 well flat bottom plate in a ratio of 10:1 with loaded or unloaded autologous (self) DCs. Wells were supplemented with 10 units/ml of IL-2 (R&D Systems) on day 0 and cultured at 37°C for six days. On day 6, unless otherwise indicated, wells were pulsed with radioactive thymidine (PerkinElmer Waltham, MA) for 6 hours then harvested using a Filtermate cell harvester (PerkinElmer,). Thymidine incorporation was quantified using a TopCount NXT scintillation counter (PerkinElmer). To calculate the stimulation index 1×10^5^ CD4+CD25- T cells were stimulated with 1×10^4^ autologous DCs loaded with survivin (DC:survivin) in a 96 well flat bottom tissue culture plate for 6 days. Proliferation for each well was determined as described above. The stimulation index for each well was calculated against T cells similarly stimulated using unloaded autologous DCs (> = 10 replicates per donor). Stimulation Index = [1×10^5^ CD4+CD25- T cells stimulated with 1×10^4^ DC:survivin (numerator)]/[Mean of > =10 DC:null stimulated T cell controls (denominator)].

### Limiting dilution analysis

The precursor frequencies of survivin-specific CD4+ T cells were determined by limiting dilution analysis as previously described [45]. Briefly, CD4+CD25- cells were seeded into 96 well plates in a two-fold descending serial dilution ranging from 100,000 cells/well to 3,125 cells/well in a flat bottom plate and 5,000 to 80 cells/well in a round bottom plate with a total of 10 replicate wells at each concentration. These cells were cocultured with a fixed number of DCs (1×10^4^ for flat bottom plates or 2×10^3^ for round bottom) which were either loaded with the survivin peptide pool (DC:survivin) or unloaded (DC:unloaded). Control wells contained the top concentration of T cells/well for that plate or DCs alone (1×10^4^ or 2×10^3^). Cells were cultured at 37°C for six days in XVIVO-15 media supplemented with 10% human AB serum (SeraCare) and 10units/ml IL-2 (R&D Systems). On day 6 [^3^H]thymidine incorporation assay was performed. The number of positive wells and total wells tested against a given peptide pool were entered into a publically available extreme limiting dilution analysis software program from Walter+Eliza Hall Bioinformatics (http://bioinf.wehi.edu.au/software/elda/). This program calculates the frequency and 95% confidence interval (95% CI) of replicating T cells. Wells were considered to be positive if cpm was greater than the mean plus three times the standard deviation of the mean of all unloaded DC control wells (10 or more replicates) at that same T cell concentration.

### IFNγ ELISA

Supernatant was collected from each well and developed using an IFNγ ELISA kit (eBiosciences, San Diego, CA) per the manufacturer’s protocol. Briefly, ELISA plates (9018, Corning Costar) were incubated overnight at 4°C with 100 μL purified anti-human IFNγ. Plates were washed and incubated with assay diluent for one hour at room temperature to block the wells from non-specific binding. Plates were washed and incubated for two hours at room temperature with cell culture supernatant or a standard curve created by performing a 2-fold serial dilution of a 500 pg/ml standard. Plates were washed and incubated at room temperature for one hour with 100 μl biotin-conjugated anti-human IFNγ. Plates were then washed again and incubated for 30 minutes at room temperature with 100 μl/well Avidin-HRP. Plates were washed and developed with 100 μl TMB substrate solution for 15 minutes. The reaction was stopped by adding 50 μl/well of 1 M phosphoric acid. The ELISA plates were analyzed at 450 nanometers using a Versamax microplate reader equipped with SoftMax Pro 5 software (Molecular Devices, Sunnyvale, CA).

### Quantitative PCR

Messenger RNA was extracted from healthy donor PBMCs or multiple myeloma patient tumor cells by Trizol reaction per the manufacturer’s protocol (Invitrogen, Grand Island, NY). mRNA was quantified and assessed for purity using a Nanodrop ND-1000 spectrophotometer (Thermo Scientific, Waltham, MA). cDNA was created using a High Capacity cDNA reverse transcription kit according to the manufacuter’s protocol (Applied Biosystems, Waltham, MA). qPCR was performed using an Applied Biosystems 7900 HT Fast Real-Time PCR system in MicroAmp optical 96-well reaction plates using Taqman universal PCR master mix and primer-probe sets for BIRC5 (survivin) and GAPDH genes (Applied Biosystems). Data were anaylyzed using SDSv2.2.2 software from Applied Biosystems. Survivin mRNA expression was normalized and reported in relationship to GAPDH mRNA expression.

### Dendritic cell transfection

Following plastic adherent generation, DCs were re-suspended in 500 μL serum- free XVIVO-15 media supplemented with GM-CSF and IL-4 and transfected with 20,000 viral particles/cell of previously described adenovirus expressing mutant full length survivin for 2 hours at 37°C [43]. After 2 hours, 2×10^5^ DCs/well were seeded into 24 well plates and supplemented with 1.5 ml of complete culture media (XVIVO-15+10% human serum (SeraCare)+Penicillin/Streptomycin) for an additional 24 hours.

### T cell expansion

2×10^6^ CD4+CD25- T cells/well isolated by magnetic bead negative selection were seeded into 24 well plates containing DCs transfected with survivin adenovirus, peptide pool loaded DCs or unloaded DCs in complete culture media (CCM) supplemented with 10units/ml IL-2 (R&D Systems). Cells were cultured for 12 days then T cells were collected, washed, counted and re-suspended in CCM without cytokines and rested for 48 hours before use in limiting dilution analysis. The fold expansion of survivin reactive T cells was determined by performing the survivin frequency assay by LDA against survivin peptide pools both before and after expansion. Live cells after expansion were enumerated by trypan blue. Fold expansion equation: Numerator = (total live CD4+ cells after expansion) X (post-expansion survivin precursor frequency); Denominator = (total CD4+CD25- cells before expansion) X (pre-expansion survivin precursor frequency).

### Immunohistochemistry

Four micrometer sections of diagnostic pathology tissue blocks from myeloma patients were stained for survivin using a Ventana Discovery XT automated system (Ventana Medical Systems, Tucson, AZ) as per manufacturer‘s protocol with proprietary reagents. The slides underwent deparaffinization on the automated system with EZ Prep solution (Ventana). Heat-induced antigen retrieval method was used in Cell Conditioning 1 (Ventana). A rabbit primary antibody to Survivin, (#NB500-201, Novus Biological, Littleton, CO) at a 1:2000 concentration in Dako antibody diluent (Carpenteria, CA.) was used. The tissues were incubated with the antibody for 60 min. The Ventana Anti-Rabbit Secondary Antibody was used as detection system (16 min). The Ventana OmniMap kit and slides were then counterstained with Hematoxylin, dehydrated and coverslipped for pathologioc evaluation. Breast carcinoma tissue was used as positive control. Negative controls were included by omitting the survivin antibody during the primary antibody incubation. The stains were interpreted by a senior pathologist using the Allred scoring system [46].

## Additional files

Additional file 1: Figure S1.
**(A)** For three healthy donors, the CD4+ T cell responses against a survivin peptide pool loaded onto self-DCs was evaluated by thymidine incorporation as described in the methods. Responses against CD4+ cells depleted of CD25+ cells were greater than that of total CD4+ cells (5 or more replicates per patient, p < 0.05 by t test). **(B)** Depletion of CD25+ cells from CD4+ cells removes CD25+CD127-, regulatory T cells. Flow plot is representative of three experiments and gated cells were also FoxP3+ by intracellular stain. **(C)** The addition of autologous CD8+ cells does not increase CD4+CD25- proliferative responses against survivin. For 3 consecutive myeloma patients, T cells were separated and stimulated with self-DCs loaded with a survivin peptide pool or DC:unloaded control. The stimulation index was calculated for CD4+CD25-(1×10^6^), CD8 (1×10^6^), or a combination of CD4+CD25-(6×10^5^) and CD8 + (4×10^5^) cells. Stimulation index = [1×10^5^ T cells stimulated with 1×10^4^ DC:survivin (numerator)/DC:null stimulated T cell controls (denominator)]. All conditions and controls performed in quadruplicate or greater wells using the same CD4/CD8 ratio. Bars represent the mean stimulation index of all 3 patients, error bars indicate standard deviation. ns = p > 0.1. Additional file 2: Figure S2.
**(A)** CD4+CD25- T cells from healthy donors (n = 11) and multiple myeloma patients (n = 7), were stimulated with self-DCs loaded with common infectious antigen peptide pools (DC:CEFT) or survivin derived peptide pools (DC:survivin), or vehicle only control. The stimulation index was calculated as described in the methods. Healthy donor and myeloma patient CD4+CD25- responses against CEFT were similar, while myeloma patient CD4+CD25- responses against survivin decreased. Proliferation by thymidine incorporation **(B)** and IFN-gamma by ELISA **(C)** were measured at both 3 days and 6 days after co-culture with DC:survivin or unloaded vehicle control. Responses at day 3 were similar for healthy donors and myeloma patients. Bars represent the mean stimulation index for all patients in that condition and error bars represent the standard error of the mean. Additional file 3: Figure S3.
**(A-C)** Adherent monocyte DCs from 4 MM patients were prepared for 6 days as described in the methods. A second condition was concurrently prepared with an additional two day cytokine maturation step (TNF-alpha, IL-1beta, IL-6 and PGE-2). DCs from each condition were loaded with peptide for 1 hour, as described. Cells were then plated as if to stimulate T cells as described. After 24–48 hours cells were collected, stained, and evaluated by flow cytometry for costimulatory markers and the MHC class II cell surface receptor, HLA-DR. Representative flow plots are shown. **(D)** The percentage of CD11c + DCs expressing CD80, CD86, and HLA-DR were the same for each group of DCs (p > 0.25 for each marker by paired T test, n = 4). Results for 3 healthy donors were similar. **(E)** The precursor frequency of naïve CD4+CD25-CD45RO- cells reactive against HIV Con gag motifs was determined for two myeloma patients, and one cord blood donor. **(F)** The survivin reactive precursor frequency, as a % of CD4+CD25- T cells, was calculated by LDA, for the peripheral blood and bone marrow of 3 myeloma patients. p=0.5 by paired non-parametric t-test.

## Abbreviations

CTL: Cytotoxic lymphocyte
HLA: Human leukocyte antigen
Treg: Regulatory T cell
LDA: Limiting dilution analysis
DC: Dendritic cell
CTV: Cell trace violet
HIV: Human immunodeficiency virus
Ad-mS: Adenoviral mutant survivin
PBS: Phosphate buffered saline
PBMC: Peripheral blood mononuclear cells
ELISA: Enzyme Linked Immunosorbant Assay
PCR: polymerase chain reaction
mRNA: Messenger RNA
IHC: Immunohistochemistry
