# Supplementary figures and images for: Survivin-specific CD4+ T cells are decreased in patients with survivin-positive myeloma
Source: J Immunother Cancer. 2015 May 19;3:20. doi: 10.1186/s40425-015-0065-1 (PMC4437443; doi:10.1186/s40425-015-0065-1)

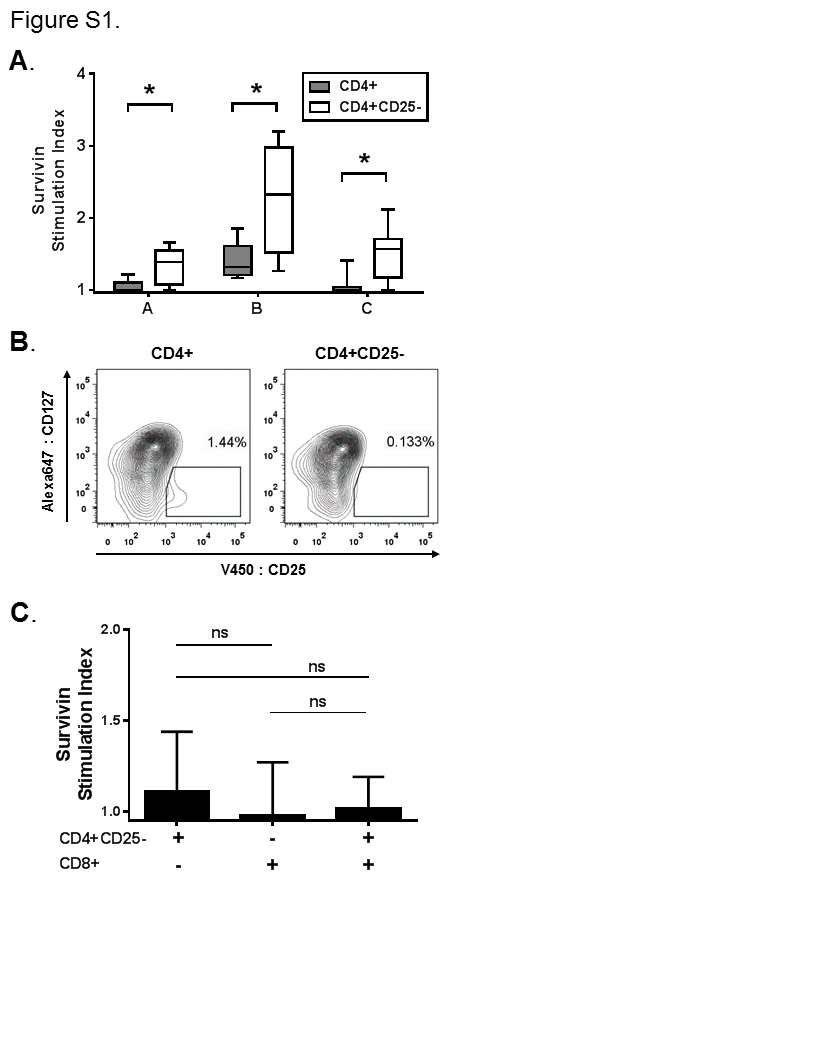

Supplement: Additional file 1: Figure S1. — (A) For three healthy donors, the CD4+ T cell responses against a survivin peptide pool loaded onto self-DCs was evaluated by thymidine incorporation as described in the methods. Responses against CD4+ cells depleted of CD25+ cells were greater than that of total CD4+ cells (5 or more replicates per patient, p < 0.05 by t test). (B) Depletion of CD25+ cells from CD4+ cells removes CD25+CD127-, regulatory T cells. Flow plot is representative of three experiments and gated cells were also FoxP3+ by intracellular stain. (C) The addition of autologous CD8+ cells does not increase CD4+CD25- proliferative responses against survivin. For 3 consecutive myeloma patients, T cells were separated and stimulated with self-DCs loaded with a survivin peptide pool or DC:unloaded control. The stimulation index was calculated for CD4+CD25-(1×10^6^), CD8 (1×10^6^), or a combination of CD4+CD25-(6×10^5^) and CD8 + (4×10^5^) cells. Stimulation index = [1×10^5^ T cells stimulated with 1×10^4^ DC:survivin (numerator)/DC:null stimulated T cell controls (denominator)]. All conditions and controls performed in quadruplicate or greater wells using the same CD4/CD8 ratio. Bars represent the mean stimulation index of all 3 patients, error bars indicate standard deviation. ns = p > 0.1. [file 40425_2015_65_MOESM1_ESM.tif]

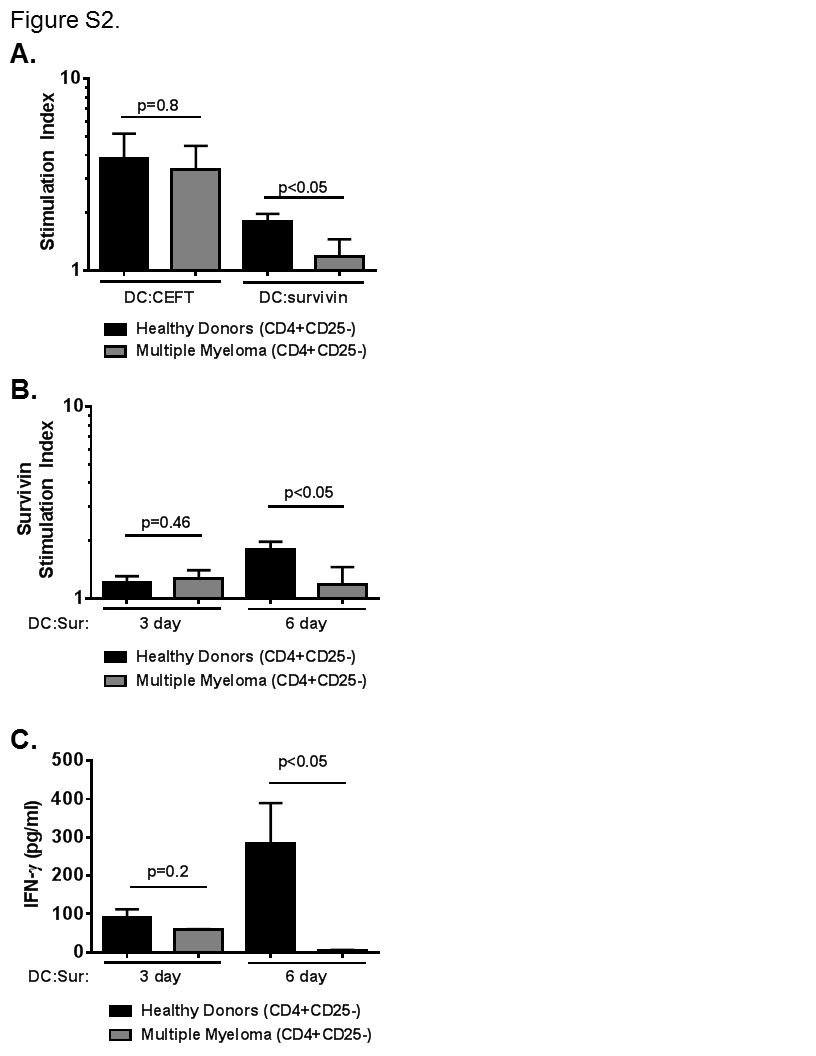

Supplement: Additional file 2: Figure S2. — (A) CD4+CD25- T cells from healthy donors (n = 11) and multiple myeloma patients (n = 7), were stimulated with self-DCs loaded with common infectious antigen peptide pools (DC:CEFT) or survivin derived peptide pools (DC:survivin), or vehicle only control. The stimulation index was calculated as described in the methods. Healthy donor and myeloma patient CD4+CD25- responses against CEFT were similar, while myeloma patient CD4+CD25- responses against survivin decreased. Proliferation by thymidine incorporation (B) and IFN-gamma by ELISA (C) were measured at both 3 days and 6 days after co-culture with DC:survivin or unloaded vehicle control. Responses at day 3 were similar for healthy donors and myeloma patients. Bars represent the mean stimulation index for all patients in that condition and error bars represent the standard error of the mean. [file 40425_2015_65_MOESM2_ESM.tif]

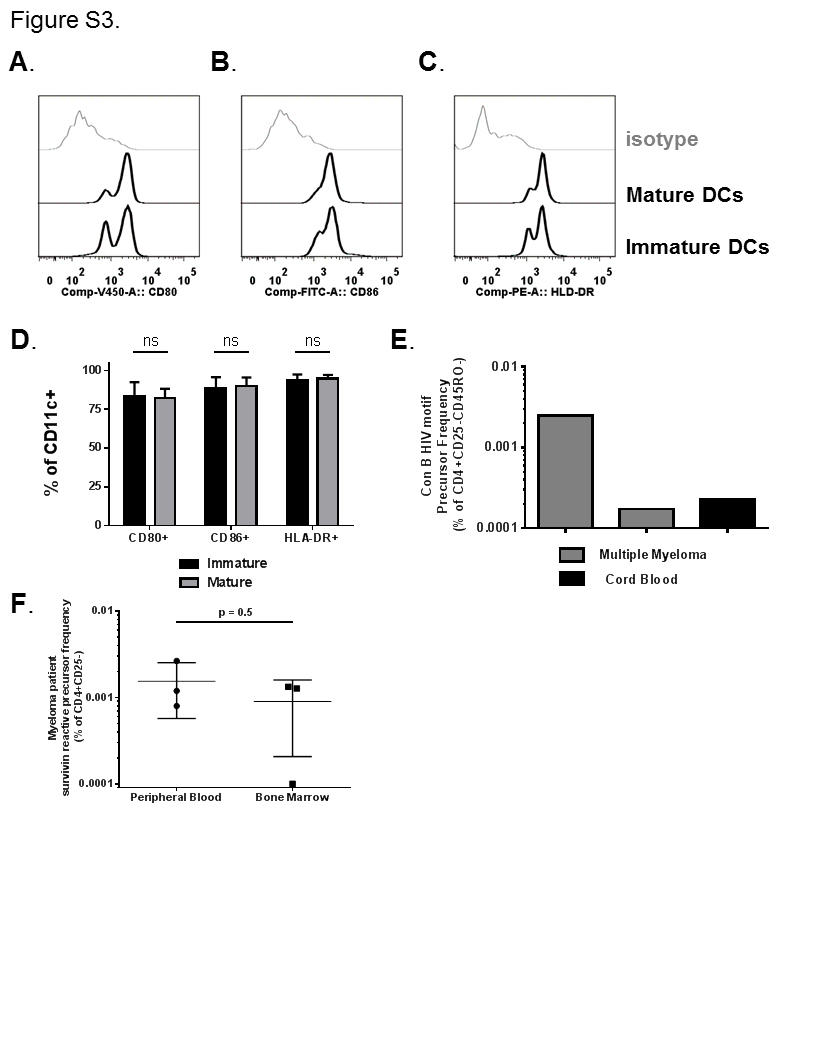

Supplement: Additional file 3: Figure S3. — (A-C) Adherent monocyte DCs from 4 MM patients were prepared for 6 days as described in the methods. A second condition was concurrently prepared with an additional two day cytokine maturation step (TNF-alpha, IL-1beta, IL-6 and PGE-2). DCs from each condition were loaded with peptide for 1 hour, as described. Cells were then plated as if to stimulate T cells as described. After 24–48 hours cells were collected, stained, and evaluated by flow cytometry for costimulatory markers and the MHC class II cell surface receptor, HLA-DR. Representative flow plots are shown. (D) The percentage of CD11c + DCs expressing CD80, CD86, and HLA-DR were the same for each group of DCs (p > 0.25 for each marker by paired T test, n = 4). Results for 3 healthy donors were similar. (E) The precursor frequency of naïve CD4+CD25-CD45RO- cells reactive against HIV Con gag motifs was determined for two myeloma patients, and one cord blood donor. (F) The survivin reactive precursor frequency, as a % of CD4+CD25- T cells, was calculated by LDA, for the peripheral blood and bone marrow of 3 myeloma patients. p=0.5 by paired non-parametric t-test. [file 40425_2015_65_MOESM3_ESM.tif]
